# Adult Intestinal Malrotation Treated with Laparoscopic Ladd Procedure

**DOI:** 10.1155/2022/6874885

**Published:** 2022-10-18

**Authors:** Noritoshi Mizuta, Takuya Kikuchi, Yoshiyuki Fukuda

**Affiliations:** Department of Surgery, Akashi Medical Center, AIJINKAI Health Care Corporation, Hyogo, Japan

## Abstract

Intestinal malrotation is a rare congenital disease caused by abnormal intestinal rotation and fixation of the intestinal tract in the early embryonic state. Adult cases are rare. A laparoscopic Ladd procedure for adult intestinal malrotation is increasingly reported, but owing to the rarity, some important aspects of the disease and its treatment may be overlooked. Three adult cases of intestinal malrotation that underwent surgery at our hospital between January 2019 and October 2020 were retrospectively examined about patient backgrounds, short-term results, and complications. All patients were male, median age was 54.6 years, and the complaints were abdominal pain and/or distention. No midgut volvulus was observed. The laparoscopic Ladd procedure was performed for all cases. One patient underwent reoperation (duodenoduodenostomy) because of impaired passage of the duodenal descending section due to postoperative pancreatic fistula. The postoperative courses of the other two patients were good. No recurrence of symptoms was observed in any of the cases. The reason for reoperation in one of the cases is considered to be pancreatic injury when the severe curve from the duodenum to the upper jejunum near the pancreatic head was straightened. Correction of the curve is important to improve passage disorder of the duodenum, but special care is required to avoid organ damage, especially during a laparoscopic procedure with forceps. The laparoscopic Ladd procedure for adult intestinal malrotation is recommended if there is no midgut volvulus; it is minimally invasive and a comparatively simple technique, but surgeons should take special care to avoid organ damage.

## 1. Introduction

Intestinal malrotation is a rare congenital disease caused by abnormal intestinal rotation and fixation of the intestinal tract from the duodenum to the transverse colon in the early embryotic stage [1]. The frequency of occurrence has been reported as 1/6000 and 1/200 of all live births, and most cases are present in the first month of life and 90% within the first year [2, 3]. The number of cases in which symptoms appear in adults is small, comprising only 0.2 to 0.5% of overall cases [3]. Various classifications of intestinal malrotation exist, but there is disparity between them. In this report, owing to its perceived simplicity, Nishijima's classification was used, classifying intestinal malrotation into the three types from a developmental point of view: nonrotation type, incomplete rotation type, and incomplete fixation type [4]. Furthermore, subtypes are presented according to the clinical type (Table 1) [4].

Ladd procedure is the generally performed treatment for intestinal malrotation. Any midgut volvulus is released; then, the abnormal band between the duodenum and the colon (Ladd's band) is resected. The base of the small intestinal mesentery including the superior mesenteric artery (SMA) is opened, followed by appendectomy and rearrangement of the intestinal tract [5]. Laparoscopic approach has become more common since the report by van der Zee and Bax [6]. In this report, the cases of three patients with adult intestinal malrotation are used to illustrate the surgical outcomes of laparoscopic Ladd procedure in our institution. The technique is safe and minimally invasive, but because there are a small number of cases, potential pitfalls require consideration.

## 2. Case Presentation

### 2.1. Case 1

**Figure 1:**
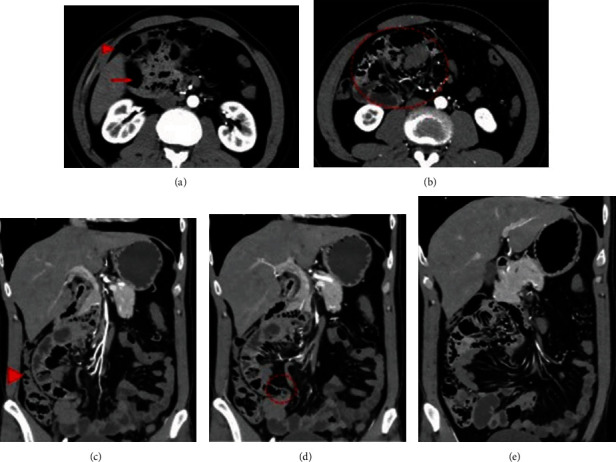
CT scan in Case 1. (a) There was no duodenal horizontal section. The duodenal descending section and jejunum (arrow) were located under the ascending colon (arrowhead). (b) The jejunum (dotted area) was located in the right upper quadrant. (c) The ascending colon (arrowhead) was recognized at the normal position. (d) The part where the jejunum excluded to the retroperitoneal side comes out into the abdominal cavity was narrowed like a “hernia defect” (dotted area). (e) The small intestine that emerges into the abdominal cavity runs to the left and then reached the ileocecal region.

A 35-year-old male felt right abdominal pain from the previous year. The pain reportedly occurred once or twice per month. Intestinal malrotation was detected on computed tomography (CT) at another hospital, but treatment was not performed. Abdominal pain did not improve, so he was referred to our hospital. CT showed absence of the duodenal horizontal section. The jejunum transitioned directly from the duodenal descending section, and both were confirmed as a mass in the right-upper abdomen under the right colonic mesentery. The colon was recognized to be in a normal position. CT showed the features of the intestinal malrotation, and the patient was admitted to our hospital on the same day (Figures 1(a)–1(e)).

**Figure 2:**
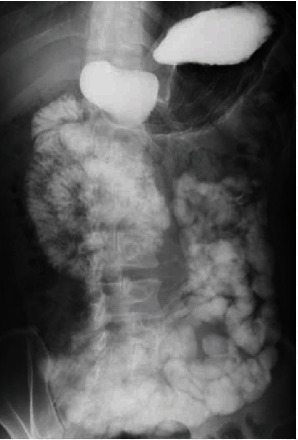
Upper GI series revealed that the duodenal descending section and the jejunum are located in the right upper abdomen, and the remaining intestine runs from the lower right abdomen to the left side.

Upper gastrointestinal (GI) series confirmed the duodenal descending section and the jejunum as a mass in the right-upper abdomen (Figure 2). Preoperative diagnosis was intestinal malrotation, and incomplete fixation (mesocolic hernia) and laparoscopic Ladd procedure were performed.

The surgery was performed by five ports under general anesthesia. The first 12 mm port was inserted from the umbilicus, and the other four ports (5 mm) comprised upper and lower parts on both sides of the abdomen.

**Figure 3:**
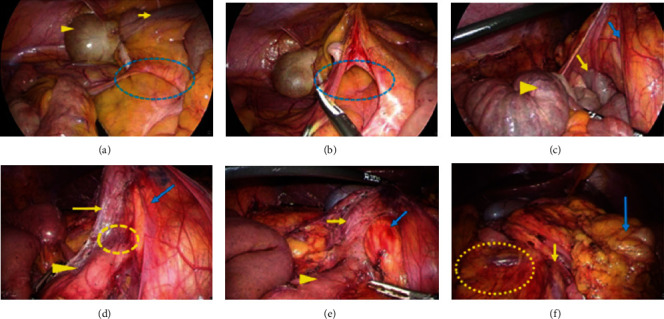
The operative findings in Case 1. (a) The small intestine run through the hernia defect (dotted area), which consists of the fibrotic adhesions between the retroperitoneum and the colon mesentery. Cecum (arrowhead) and terminal ileum (arrow) were recognized over the hernia gate. (b) The hernia defect (dotted area) was dissected. (c) The outside adhesion of the right colon and the abnormal retroperitoneal bands was also dissected. After that, the duodenum (yellow arrow) and the jejunum (yellow arrowhead) were recognized under the ascending colon (blue arrow). (d) There was a severe curve (dotted area) at the transition from the proximal side of the duodenum (yellow arrow) to the distal side (yellow arrowhead), and a membrane structure ran across the duodenum and the pancreas head (blue arrow). (e) The transition between the proximal side of the duodenum (yellow arrow) and the distal side (yellow arrowhead) was straightened. The bleeding was recognized at the pancreatic head (blue arrow). (f) The small intestinal mesentery (dotted area) was recognized at the right side, and the transverse colon (blue arrow) was at the left side. The root of the small intestinal mesentery (yellow arrow) was fully widened.

In operative findings, the small intestine was recognized under the abnormal adhesive retroperitoneal band and the right colon mesentery. The duodenum and the jejunum were released from the mesenteric pressure after dissection of the band by laparoscopic coagulating shears (LCS). The outside adhesion of the right colon was all resected by LCS, and the small intestine excluded on the dorsal side came out from the thickened tissue at the right-lower abdomen which had formed between the colon mesentery and the retroperitoneum. The thickened tissue was like “hernia defect,” and it was resected. The narrowed base of the small intestinal mesentery including SMA was widely opened, and appendectomy was performed. Finally, the small intestine was replaced at the right side, and the colon was at the left side. No fixation of the intestine to the retroperitoneum was performed (Figures 3(a)–3(f)).

**Figure 4:**
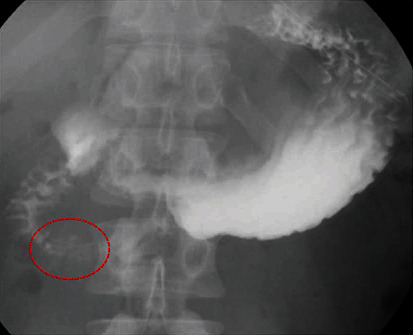
Upper GI series revealed a passage disorder of the duodenal descending portion (dotted area).

Meals were started on postoperative day (POD) 2, although vomiting occurred on POD 5. A nasal gastric (NG) tube was inserted. On POD 6, upper GI series was performed and passage disorder of the duodenum was observed (Figure 4).

**Figure 5:**
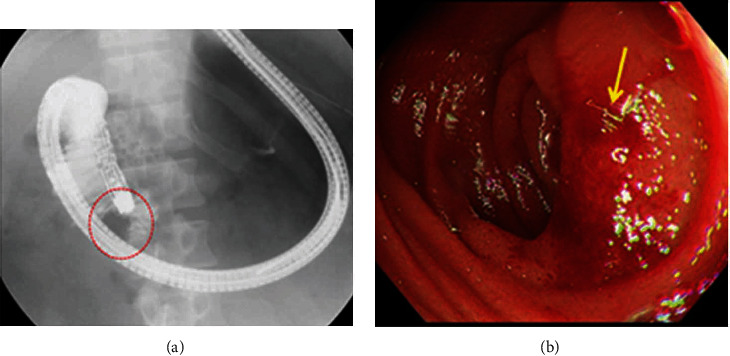
EGD findings in Case 1 on POD15. (a) EGD could not pass through the duodenal descending portion (dotted area). (b) Extrinsic compression with redness (arrow) was seen at the duodenal descending portion.

Paralytic duodenal peristalsis was considered to be the reason for the passage disorder, so conservative treatment was performed. However, 1 or 2 L of enteric content from the NG tube was continuously observed every day. Esophagogastroduodenoscopy (EGD) was performed on POD 15. The scope could not pass through the duodenum because of the external compression (Figures 5(a) and 5(b)). A conservative treatment was judged to be insufficient, and reoperation was performed on POD 16.

During reoperation, there was stenosis of the duodenal descending section. A local saponification was observed around it. The duodenal passage was judged to be impaired due to pancreatic fistula. The stenosis and the inflammation were very severe, so lysis was not thought to be possible. The proximal and distal sides of the stenosis were therefore anastomosed by Albert-Lembert method (side-to-side duodenoduodenostomy). The postoperative course was good, and the patient was discharged on POD 32.

### 2.2. Case 2

A 71-year-old male underwent appendectomy for acute appendicitis at fourteen years of age. Eight months prior to the current presentation, he had abdominal pain for the first time after a meal which continued intermittently thereafter. When the same symptom appeared to worsen, he visited the emergency department of our hospital. CT and upper GI series revealed similar results to those of Case 1. The patient was admitted to our hospital on the same day. Preoperative diagnosis was the same as Case 1, and surgery was performed. The operative procedure was almost the same as that in Case 1. The patient was discharged on POD 10.

### 2.3. Case 3

A 58-year-old male had right abdominal pain after meals over a ten-year period. He went to other hospitals, but there was no accurate diagnosis. From two months before visiting our hospital, epigastric pain and appetite loss appeared, and he lost 4 kg in weight. The pain got worse, so he visited the emergency department of our hospital. The findings of CT and upper GI series were similar to those of Cases 1 and 2. Under the same diagnosis, surgery was performed. The operative findings and procedure were almost the same as those in Cases 1 and 2. The patient was discharged on POD 7.

## 3. Discussion

In our cases, all three patients were male with a median age of 54.6 years. The period of complaints ranged between eight months and ten years. There was no midgut volvulus, and a laparoscopic Ladd procedure was performed for all cases. The postoperative courses were good, with the exception of Case 1.

Intestinal malrotation, especially in adults, is rare [1, 3], so a single surgeon is likely to encounter only a small number of surgical cases. Surgeons may never experience it. In these cases, however, we saw a relatively large number of cases within a short period of time.

The treatment for intestinal malrotation is generally the Ladd procedure [5]. Important considerations regarding Ladd procedure include the following: release of any midgut volvulus, resection of the abnormal adhesive retroperitoneal band (Ladd's band) between the duodenum and the right colon mesentery, opening the base of small intestinal mesentery including the SMA, performing prophylactic appendectomy, and rearranging the intestinal tract [5]. We suggest that resection of the abnormal band relieves compression of the duodenum or jejunum. The opening of the base of the small intestinal mesentery is especially important to create sufficient space for the small intestine and the colon, removing one of the causes of malrotation.

In our cases, the clinical symptoms were thought to have been caused by the duodenum and the jejunum being compressed by the abnormal retroperitoneal band and the right colonic mesentery. The band is connected to the adhesion between the prepancreatic fascia and the colonic mesentery. There is also a severe curve between the proximal side and the distal side of the duodenal descending portion because of the adhesive compression. This adhesion is considered to be more severe in adult cases than in infant cases because of the longer period of illness. The curve otherwise remains the same; the duodenal passage disorder does not completely improve. Not only resection of the abnormal band but also straightening of the curve is therefore necessary. It is important to perform this resection and straightening carefully in order to avoid organ damage, such as to the duodenum or the pancreas. Resection of the abnormal band and straightening the curve were performed in all of three cases. In Case 1, however, postoperative stenosis of the duodenal descending section occurred because of pancreatic fistula. The pancreas head was thought to be damaged when resecting the adhesion between the prepancreatic fascia and the right colon mesentery. In operative findings, the bleeding was recognized at the pancreatic head (Figure 3(e)). Two factors were considered to be the cause: misdiagnosis of the border of the prepancreatic fascia and the right colon mesentery and rough use of laparoscopic forceps. To avoid misdiagnosis of the border, it would be useful to confirm the pancreatic head firstly after dissecting the omentum and opening the omental bursa from the left side. It would also be useful to sufficiently replace the colon at the left side of the abdomen. In order to avoid the second factor, great care is required in performing the delicate procedure.

Opening the base of the small intestinal mesentery is also thought to be important. The narrow base of the small intestinal mesentery could lead to the patient having subsequent midgut volvulus and obstruction with potential vascular catastrophe [7]. The frequency of adult intestinal malrotation present with midgut volvulus was reported in only 15% of all adult cases [3], and prognosis for the cases of acute abdomen with midgut volvulus was very poor [8, 9]. Opening the base of small intestinal mesentery is therefore crucial to reducing the likelihood of midgut volvulus [10].

Due to the widespread use of laparoscopic surgery, there has been an increase in reports of laparoscopic Ladd procedure for intestinal malrotation. Owing to its rarity, adult cases are almost always reported within case reports, but there have been several retrospective studies for large numbers of laparoscopic Ladd procedure for adults [10–12]. Matzke et al. compared an open group (*n* = 10) and a laparoscopy group (*n* = 11); three patients in the laparoscopy group were converted to open surgery [10]. All patients with midgut volvulus underwent laparotomy [11]. The laparoscopic group resumed oral intake earlier than the open group and had a shorter length of hospital stay (LOS), and operation times were longer in the laparoscopic group [11]. No patients required reoperation in either of the groups [11].

Seymour and Anderson reported seven cases of laparoscopy; this included no cases of laparotomy but three cases with midgut volvulus [12]. No patients had postoperative complications, and symptoms were improved by the surgery.

Fraiser et al. compared laparotomy (*n* = 13) and laparoscopy (*n* = 9); three patients in the laparoscopy group were converted to open surgery [10]. In the laparotomy group, many patients had acute onset and higher American Society of Anesthesiologists Classification Score (ASA-PS) [10]. There were no significant differences in complication rates, need for reoperation, or symptom resolution, but the decrease in LOS was significant in the laparoscopy group compared with the laparotomy group [10]. Three patients needed reoperation, and two of them (one laparotomy, one laparoscopy) underwent duodenoduodenostomy due to the stenosis of the descending part of the duodenum similar to our case [10]. One of them required reoperation several weeks after the laparoscopic Ladd procedure and was perioperatively found to have significant retroperitoneal adhesions on operative findings [10]. The reason for the adhesion occurring by pancreatic fistula was unclear.

In conclusion, a laparoscopic Ladd procedure for adult intestinal malrotation is recommended because it is minimally invasive and has shorter LOS compared with laparotomy. However, in an emergent case, such as with intestinal necrosis or midgut volvulus, laparotomy should instead be selected. Careful technique is required due to the risk of organ damage.

## Figures and Tables

**Table 1:** The classification of intestinal malrotation (based on Nishijima's literature [4]).

(1) Nonrotation
(2) Incomplete rotation
(a) Incomplete rotation of both^∗^ limbs
(b) Incomplete rotation of duodenojejunal limb (extrinsic obstruction of the duodenum)
(c) Nonrotation of the cecocolic limb
(d) Reverse rotation
(3) Incomplete fixation
(a) Mesocolic hernia (paraduodenal hernia, mesenteric hernia)
(b) Mobile cecum

^∗^Duodenojejunal limb and cecocolic limb.
